# 

**Published:** 1858-05

**Authors:** 


MAY CONTENTS OF "HALL'S JOURNAL OF HEALTH," NEW YORK."

*One Dollar a Year; Specimen-Numbers, Ten Cents.*

Family Order.................... 101
New Potato..................... 104
Human Growth. . .............. 104
Poisonous Milk.................. 105
Sleep Delicious.................. 108
Teeth of Children................ 109
Our Troubles.................... 110
A Chat about Health............ 115
Relapses.......................... 116
Going Down...................... 117
Sensible Gentleman............. 118
Doctors........................... 119
Recreations...................... 120
Cancer........................... 121
Dyspeptic Correspondence....... 123
Fruits of the Earth.............. 123
Eating and Exercise............. 124

